# Sodium Oxybate: a Substitute for Alcohol?

**DOI:** 10.1192/j.eurpsy.2022.144

**Published:** 2022-09-01

**Authors:** A. Gual, H. López-Pelayo, P. Bruguera, L. Miquel

**Affiliations:** 1 Centre Bonanova, Psychiatry, Barcelona, Spain; 2 Hospital clinic, Addictions Unit, Barcelona, Spain

**Keywords:** sodium oxybate, relapse prevention, Alcohol Treatment

## Abstract

Gamma-hydroxybutyrate (GHB) is a neurotransmitter found naturally in the human brain. Sodium oxybate (SO) is the sodium salt of GHB. In 2000 GHB was classified a Schedule I controlled substance, while SO became a Schedule III controlled substance for medicinal use under the Controlled Substances Act. SO and alcohol share a similar pharmacological profile. GHB acts on GABA_B_ receptors and extrasynaptic GABA_A_ receptors resulting in alcohol-mimetic effects in several CNS actions. It substitutes the discriminative stimulus effects of alcohol in rats, and has cross tolerance with alcohol. All together, this leads to think of SO as a substitution therapy for alcohol use disorders. SO was initially studied in the prevention of alcohol withdrawal, and it showed similar efficacy to benzodiacepines. The studies on relapse prevention were developed later and the results are mixed and more complex to understand. While open label studies show a positive effect, RCTs have not been able to show a significant effect for the whole sample. Nevertheless, post-hoc analysis show a robust effect in the subsample of patients with high risk drinking levels, that would be the preferred target for a substitution treatment. The potential for abuse of GHB is well documented, which should be no surprise for a substitution treatment. Nevertheless, when correctly prescribed the risk of abuse of SO remains very low, as shown both in clinical trials and in the pharmacovigilance database, with more than 260000 cases documented. SO can be considered a substitution treatment, effective in patients with high risk drinking levels.

**Disclosure:**

I was investigator in the SMO study on sodium oxybate, funded by D&A Pharma.

